# Facial neuromuscular junctions and brainstem nuclei are the target of tetanus neurotoxin in cephalic tetanus

**DOI:** 10.1172/jci.insight.166978

**Published:** 2023-06-08

**Authors:** Federico Fabris, Stefano Varani, Marika Tonellato, Ivica Matak, Petra Šoštarić, Patrik Meglić, Matteo Caleo, Aram Megighian, Ornella Rossetto, Cesare Montecucco, Marco Pirazzini

**Affiliations:** 1 Department of Biomedical Sciences, University of Padua, Padua, Italy.; 2 Department of Pharmacology, School of Medicine, University of Zagreb, Zagreb, Croatia.; 3 Padua Neuroscience Center, University of Padua, Padua, Italy.; 4 Institute of Neuroscience, National Research Council, Padua, Italy.; 5 Interdepartmental Research Center of Myology (CIR-Myo), University of Padua, Padua, Italy.

**Keywords:** Neuroscience, Neurological disorders, Neuromuscular disease, Toxins/drugs/xenobiotics

## Abstract

Cephalic tetanus (CT) is a severe form of tetanus that follows head wounds and the intoxication of cranial nerves by tetanus neurotoxin (TeNT). Hallmarks of CT are cerebral palsy, which anticipates the spastic paralysis of tetanus, and rapid evolution of cardiorespiratory deficit even without generalized tetanus. How TeNT causes this unexpected flaccid paralysis, and how the canonical spasticity then rapidly evolves into cardiorespiratory defects, remain unresolved aspects of CT pathophysiology. Using electrophysiology and immunohistochemistry, we demonstrate that TeNT cleaves its substrate vesicle-associated membrane protein within facial neuromuscular junctions and causes a botulism-like paralysis overshadowing tetanus spasticity. Meanwhile, TeNT spreads among brainstem neuronal nuclei and, as shown by an assay measuring the ventilation ability of CT mice, harms essential functions like respiration. A partial axotomy of the facial nerve revealed a potentially new ability of TeNT to undergo intra-brainstem diffusion, which allows the toxin to spread to brainstem nuclei devoid of direct peripheral efferents. This mechanism is likely to be involved in the transition from local to generalized tetanus. Overall, the present findings suggest that patients with idiopathic facial nerve palsy should be immediately considered for CT and treated with antisera to block the potential progression to a life-threatening form of tetanus.

## Graphical abstract

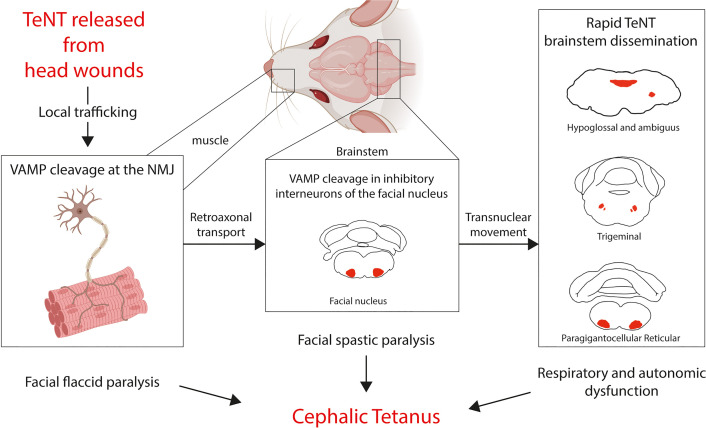

## Introduction

Tetanus neurotoxin (TeNT) is a 150 kDa protein released by *Clostridium tetani* during infections of necrotic wounds, which causes a life-threatening neuroparalytic syndrome characterized by tonic muscle contractions and painful muscle spasticity (1–3).

Tetanus pathogenesis begins with the entry of TeNT into peripheral nerve terminals followed by retroaxonal transport and release into the spinal cord and brainstem (4–8). Therein, the toxin enters the synaptic terminals of inhibitory interneurons via synaptic vesicle endocytosis (9) and translocates its catalytic metalloprotease domain in the presynaptic cytosol (10), where it cleaves a single-peptide bond of vesicle-associated membrane protein (VAMP) (11). This biochemical lesion disrupts the molecular machinery responsible for synaptic vesicle fusion, with the presynaptic membrane inhibiting the release of inhibitory neurotransmitters, which in turn leads to motoneuron overexcitability and muscle spastic paralysis (5).

Tetanus can be effectively prevented via vaccination with a formalin-inactivated TeNT (tetanus toxoid) or by passive immunization with anti-TeNT immunoglobulins, which is the prophylactic therapy used with patients presenting in the emergency room with necrotic skin wounds and uncertain vaccination status (3, 8, 12, 13). Nonetheless, tetanus remains a major killer in low-income countries, where vaccination and antisera availability are limited, and where the disease affects particularly newborns in the tremendous form of *tetanus neonatorum* (12–15). Novel research for inexpensive chemical inhibitors of TeNT should be encouraged (16).

The spastic paralysis of tetanus starts from the face with lockjaw (trismus), distortion of mouth and eyes (risus sardonicus), followed by neck stiffness and trunk arching (opisthotonos). Spasticity progresses in a descending manner and eventually affects all muscles, causing body exhaustion and patient death by a cardiorespiratory deficit (17). When a limited amount of TeNT is released in a confined anatomical area, a local form of tetanus develops with the involvement of regional muscles. This disease can then evolve into generalized tetanus depending on the further release of TeNT (2).

A rare (about 3% of cases), yet particularly dangerous, form of tetanus is cephalic tetanus (CT), which develops from infections of craniofacial wounds, of the inner ear, or of mouth gingivae with *C*. *tetani* spores. CT begins with a peculiar botulism-like cranial nerve palsy that generally precedes, or sometimes accompanies, trismus and risus sardonicus (17–19). This unusual manifestation complicates the diagnosis of tetanus, which often goes unsuspected for days, causing an unfavorable delay in the pharmacological intervention. For this reason, CT is a form of tetanus accompanied by a poor prognosis (17) because CT patients can rapidly develop cardiorespiratory deficits before, or even without, generalized spasticity (20–22).

How TeNT causes overlapping flaccid and spastic paralysis and how this can then rapidly evolve into cardiorespiratory defects remain unresolved aspects of CT pathophysiology.

Using a rodent model of CT based on the local injection of TeNT in the whisker pad and the use of an antibody that recognizes with high specificity TeNT-cleaved VAMP, but not intact VAMP (23), we show here that CT facial palsy is caused by the TeNT-mediated proteolysis of VAMP within the neuromuscular junctions (NMJs) of facial muscles. This action precedes and then overlaps with the canonical spastic paralysis ascribed to the TeNT activity within inhibitory interneurons of the spinal cord. We also report that specific nuclei of the brainstem are affected in CT and that TeNT can spread to other brainstem nuclei controlling critical functions, including mastication, deglutition, and respiration, via both peripheral diffusion and intraparenchymal dissemination of the toxin. These findings explain why CT can rapidly evolve into a life-threatening form of tetanus and suggest that patients presenting a facial nerve palsy of unknown origin should be immediately considered for CT and treated with the effective injection of human anti-TeNT immunoglobulins.

## Results

### TeNT local injection in the mouse whisker pad recapitulates human CT.

**Figure 1:**
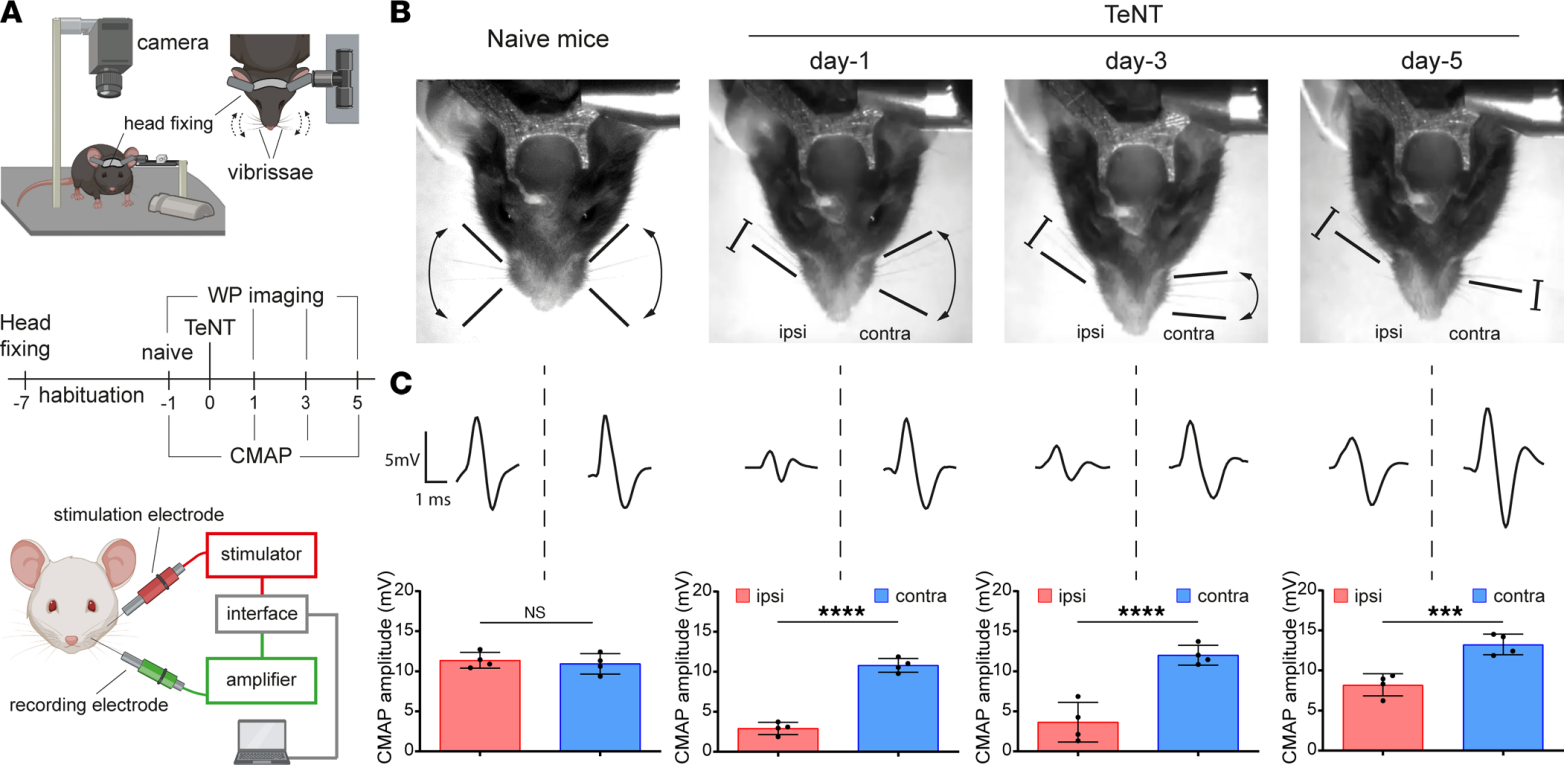
TeNT causes a botulinum neurotoxin–like flaccid paralysis upon injection in the WP in a model of CT. (**A**) The top panel illustrates the experimental setup to video record WP activity in head-fixed mice in a model of CT upon TeNT injection in the WP (1 ng/kg in a final volume of 1 μL) as a model of CT. Mice are held at the center of a mouse arena through a metal bar cemented to the skull; an infrared camera is positioned on top of the mouse snout to record the whisking activity. The bottom panel schematizes the apparatus to measure the compound muscle action potential (CMAP): the green electrode records WP myofibers’ depolarization elicited by facial nerve stimulation through the red electrode; stimulation and signal amplification are controlled with a computer connected via an interface. The central panel shows the time course of a typical experiment for WP video recording and CMAP analysis across TeNT injection in the WP. (**B**) Representative video frames showing the whisking ability in naive mice and at indicated time points after TeNT inoculation in injected (ipsi) and noninjected (contra) WPs; black arrows and bars indicate the movement ability of the vibrissae as deduced from recorded videos; segments with blunt ends indicate full paralysis. (**C**) Representative traces of CMAP recordings in ipsi and contra WP (top) and their quantification (bottom) at indicated times after TeNT injection. Data are expressed as means ± SD; ***P < 0.001, ****P < 0.0001 assessed by Student’s *t* test. Black circles indicate the number of animals used in the experiment.

To study CT pathophysiology, we established an experimental model in rodents based on the local injection of TeNT into the whisker pad (WP), the group of muscles responsible for vibrissa movement in whisking animals. The WP receives sensorimotor innervation from the facial nerve, and its neuromuscular activity can be recorded via live imaging of vibrissae in head-fixed animals (24) and by compound muscle action potential (CMAP) electromyography in anesthetized animals (25). Both techniques allow the monitoring of WP activity with time, which offers the advantage of evaluating TeNT effects in the same animal before and after toxin inoculation (Figure 1A). While naive mice freely moved their vibrissae, covering a wide angle depending on the whisking activity, TeNT-treated mice progressively lost the ability to move the ipsilateral WP, and vibrissae bent toward the jaw, appearing fully paralyzed after 1 day, a condition persisting also at days 3 and 5 (Figure 1B and Supplemental Videos 1–4; supplemental material available online with this article; https://doi.org/10.1172/jci.insight.166978DS1). Conversely, contralateral vibrissae were normal on day 1 but progressively stacked around their position, appearing paralyzed by day 5, though differently from ipsilateral ones. To characterize the 2 types of paralysis, we assessed neurotransmission at the NMJ by CMAP electromyography (Figure 1C). Facial nerve stimulation in naive mice elicited CMAP displaying a biphasic trace in both WPs, while TeNT provoked a marked reduction of maximal CMAP amplitude in the ipsilateral WPs at all time points, an indication of defective neurotransmitter release at the NMJ suggestive of flaccid paralysis (Figure 1C). To further test this possibility, we compared the TeNT-induced paralysis with that caused by botulinum neurotoxin type B (BoNT/B), another clostridial neurotoxin long known to cause flaccid paralysis by cleaving VAMP at the NMJ at the same peptide bond cleaved by TeNT (11, 23). In head-fixed mice, BoNT/B injection elicited a paralysis of the vibrissae that closely resembled the one caused by TeNT (Supplemental Video 5), and, consistently, the CMAP electromyography showed a strong decrease in amplitude (Supplemental Figure 1). Interestingly, the decrease in CMAP amplitude caused by TeNT recovered by day 5, indicating that this paralytic effect is rapidly reversible in mice. At the same time, contralateral WPs displayed no changes in CMAP traces and amplitude as occurred in naive mice, indicating that TeNT produces its local effect only in injected muscles.

Together, these results suggest that the action of TeNT at the NMJ is similar to the one of botulinum neurotoxins, does not cause degeneration of the motor axon terminals or the death of the motoneurons, and is rapidly reversed (8, 26, 27). This botulism-like paralysis in injected muscles is then followed, in a few hours, by the canonical spastic paralysis of other head muscles, found here in the contralateral WP muscle. These findings are reminiscent of what occurs in human CT in patients manifesting simultaneously a flaccid and spastic paralysis of facial muscles (please see ref. 19 for a direct comparison), thus qualifying this mouse model for the study of the molecular pathogenesis of CT.

### CT flaccid paralysis is caused by the TeNT-mediated cleavage of VAMP within motor axon terminals of facial NMJs.

**Figure 2:**
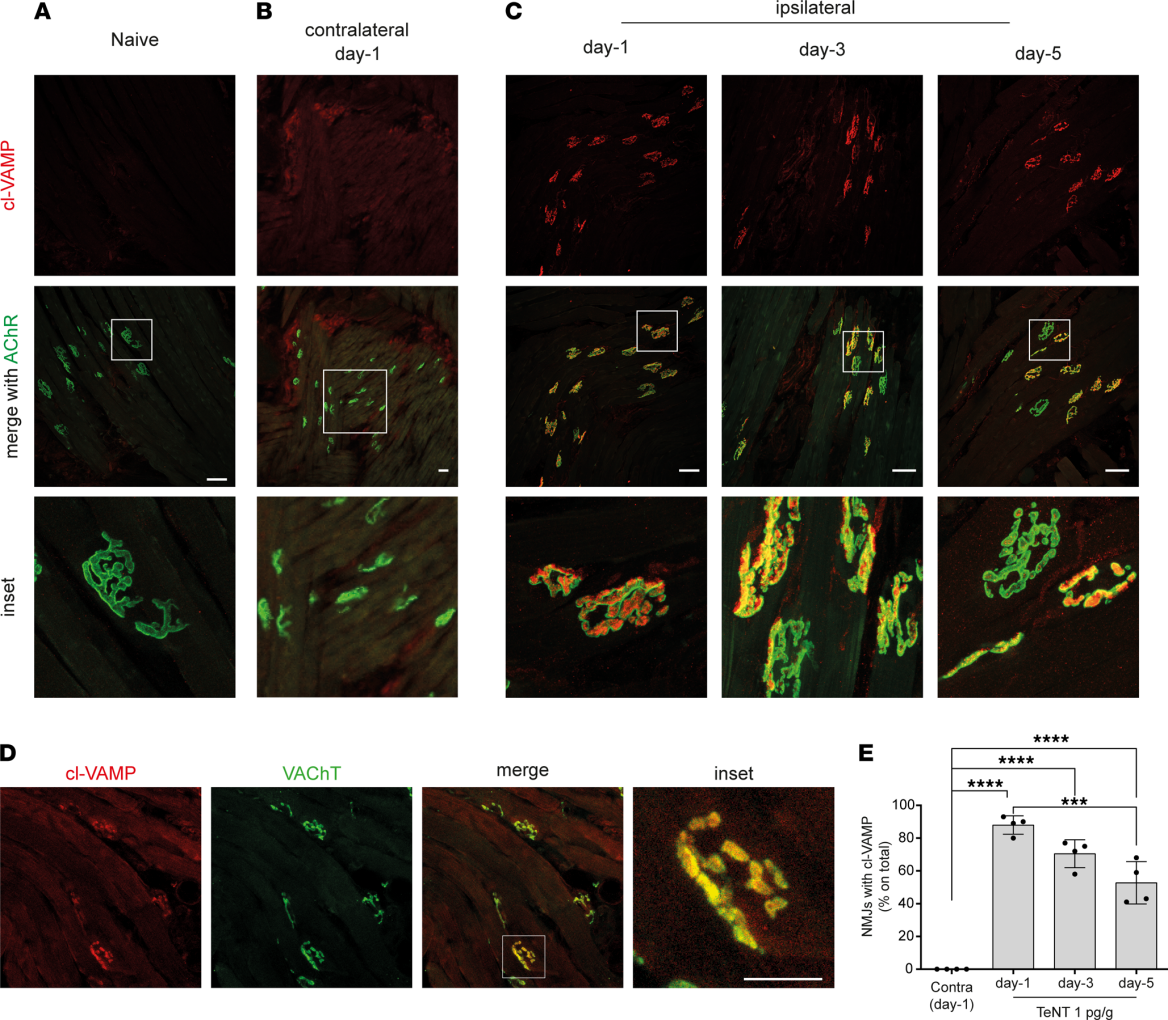
TeNT cleaves its target VAMP at motor axon terminals of the WP in mice. Confocal images of WP musculature from (**A**) naive and TeNT-treated mice (**B**) contralateral or (**C**) ipsilateral to injection at indicated times after injection; the red signal indicates the cleavage of VAMP at the NMJ identified through the labeling of nicotinic acetylcholine receptors (AChR, shown in green) with fluorescent α-bungarotoxin; insets show a 5× original magnification (**A** and **C**) and 3× original magnification in **B**. (**D**) Confocal images showing colocalization between cl-VAMP (red) and the vesicular transporter of acetylcholine (VAChT, green), a protein marker of synaptic vesicles, as expected from TeNT cleavage of VAMP on synaptic vesicles at the motor axon terminal; scale bar, 50 μm. (**E**) Quantification reporting the percentage of NMJs positive for the signal of cl-VAMP in the ipsilateral WP at indicated time points after TeNT injection compared with the contralateral at day 1. Data are expressed as means ± SD; ***P < 0.001; ****P < 0.0001 assessed by 1-way ANOVA with Bonferroni’s test. Black circles indicate the number of animals used in the experiment.

Based on CMAP findings, we hypothesized that CT nerve palsy could derive from the direct activity of TeNT at the NMJs of the WP muscle. To test this possibility, we isolated the ipsilateral and contralateral WPs at different time points after TeNT injection and stained the muscles with an antibody that specifically recognizes VAMP only after TeNT proteolysis (hereafter indicated as cl-VAMP), not before cleavage (23). The postsynaptic membrane of the NMJs was stained with fluorescent α-bungarotoxin, which binds tightly to nicotinic acetylcholine receptors (AChRs). WPs injected with saline did not show cl-VAMP staining (Figure 2A), similarly to WPs contralateral to the injection side, throughout the entire course of TeNT intoxication (Figure 2B and Supplemental Figure 2A). Conversely, a clear staining of cl-VAMP appeared in the ipsilateral WPs (Figure 2C). This signal was localized within presynaptic terminals and associated with synaptic vesicles, as indicated by its colocalization with the vesicular acetylcholine transporter (VAChT), a protein marker of these organelles (Figure 2D). Consistent with the time course of CMAP amplitude, NMJ staining quantification showed that the number of cl-VAMP–positive synapses peaked at day 1 and then gradually decreased with time (Figure 2E).

To monitor the correlation between VAMP cleavage and flaccid paralysis, a dose dependence study was performed by injecting increasing doses of TeNT. A dose of 0.25 pg/g did not cause evident cleavage of VAMP at the NMJ (Supplemental Figure 2, B and C), and, consistently, CMAP amplitude was not altered (Supplemental Figure 2D), indicating that TeNT did not cause flaccid paralysis at this dosage. At the same time, injected animals developed spastic paralysis about 2 days after injection. At 0.5 pg/g, TeNT caused a VAMP cleavage lower than the one obtained with 1 pg/g. In parallel, CMAP showed an intermediate decrease in amplitude, indicating that there is a correlation between VAMP cleavage at the NMJ and TeNT-induced flaccid paralysis. Together with the progressive loss of cl-VAMP staining accompanying the functional recovery at day 5, these results also suggest that the reversible nature of TeNT paralysis at the NMJ depends on the degradation of the TeNT light chain within axon terminals and turnover of cl-VAMP, as reported for the other botulinum neurotoxins cleaving different soluble NSF attachment protein receptor (SNARE) proteins (27–29).

**Figure 3:**
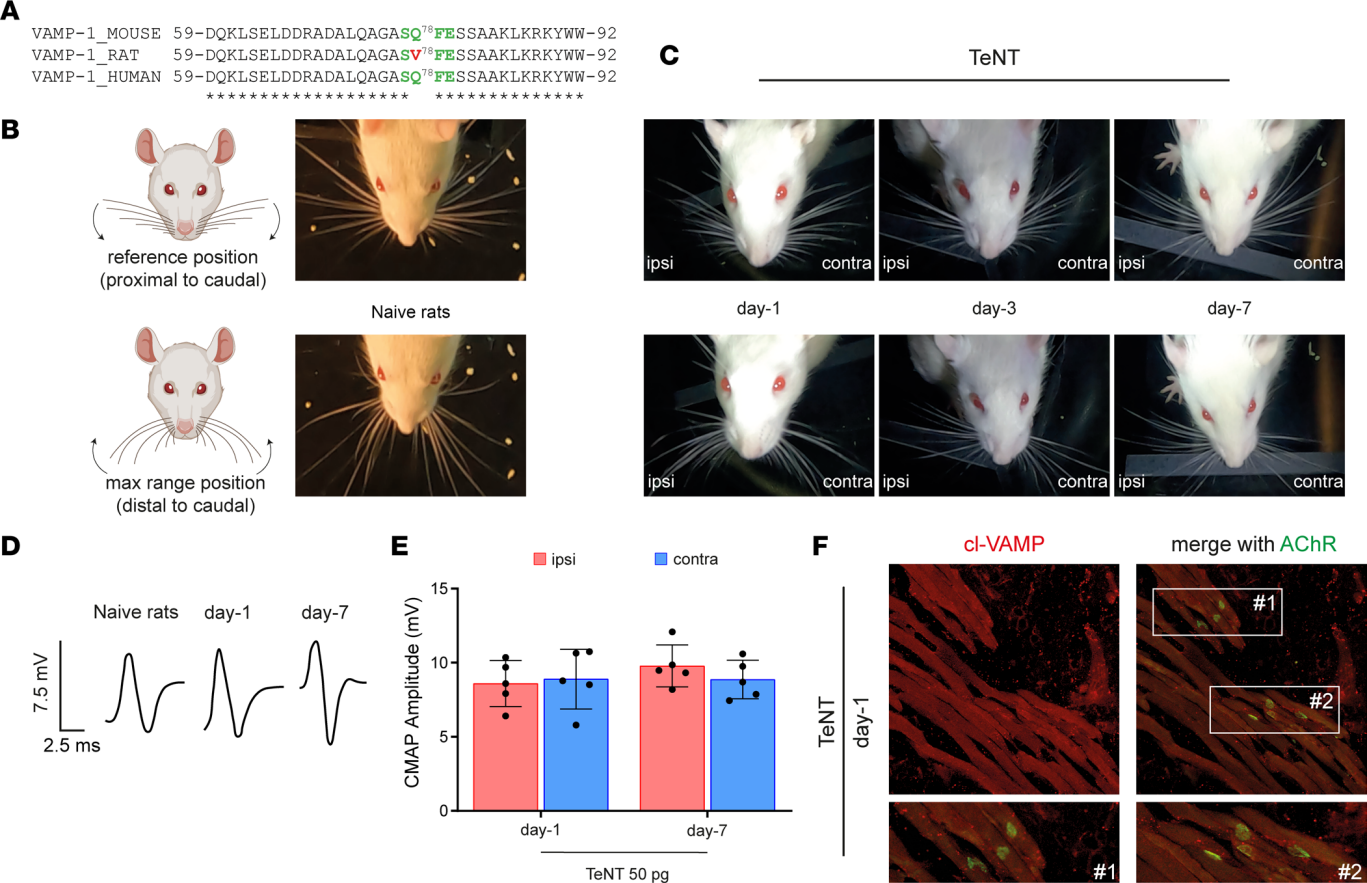
A point mutation in VAMP-1 renders rats resistant to TeNT peripheral neuroparalysis. (**A**) Alignment showing the peptide bond cleaved by TeNT (green) in mouse and human VAMP-1 that is mutated in rats, making the protein resistant to cleavage. (**B**) Scheme showing the extensions of vibrissae in rats used to evaluate their whisking behavior through video recording after unilateral TeNT injection; top and bottom panels show the maximum extensions proximally and distally from the rat snout; arrows indicate the direction of vibrissa movement. (**C**) Representative video frames from naive and TeNT-treated rats at the indicated time points after TeNT injection (50 pg in total in a final volume of 10 μL) in the ipsilateral WP; top and bottom panels show that at day 1 ipsilateral whisking is normal with no flaccid paralysis, while vibrissae are stacked around their position at day 3 and day 7, suggestive of WP spastic paralysis. (**D**) Representative traces of CMAP recordings at the indicated time points after the injection of TeNT in the WP and (**E**) their quantification. Data are expressed as means ± SD. Black circles indicate the number of animals used in the experiment. (**F**) Confocal images of the ipsilateral WP musculature 1 day after TeNT injection; the lack of cl-VAMP immunostaining indicates no TeNT activity at the NMJ identified through AChR labeling (green) with fluorescent α-bungarotoxin; insets show a 3× original magnification.

To provide further evidence that VAMP cleavage causes TeNT-induced flaccid paralysis, we extended the experiment to rats. This species carries a point mutation at the cleavage site of VAMP-1, rendering it resistant to TeNT proteolysis (Figure 3A). This is an effective biochemical knockin model (23, 30). Rats have long vibrissae whose movements can be simply and easily monitored via video recording with a high-speed camera (Supplemental Video 6). We examined their movements from proximal and distal positions with respect to the caudal part of the body (cartoons of Figure 3B). These 2 positions were identified both in naive rats (Figure 3B) and in injected rats on day 1 (Figure 3C and Supplemental Video 7), suggesting that vibrissae movements did not display obvious alterations in both injected and noninjected WPs. At variance, ipsilateral whiskers began to remain stacked in between the distal and proximal positions on day 3 and appeared fully paralyzed by day 5 (Figure 3C and Supplemental Videos 8 and 9). To discriminate whether paralysis was flaccid or spastic, we performed a CMAP analysis. Both injected and contralateral WPs displayed a normal neuromuscular transmission, indicating a spastic paralysis (Figure 3, D and E). Consistently, we failed to detect the staining of cl-VAMP in the motor axon terminals of injected WPs (Figure 3F).

Together, these results show for the first time to our knowledge that TeNT can cleave VAMP in the cytosol of peripheral motor axon terminals, causing (in susceptible species) a reversible flaccid paralysis similar to that caused by botulinum neurotoxins.

### The peripheral effect of TeNT at the NMJ is dominant on its central activity within inhibitory interneurons in the FN.

**Figure 4:**
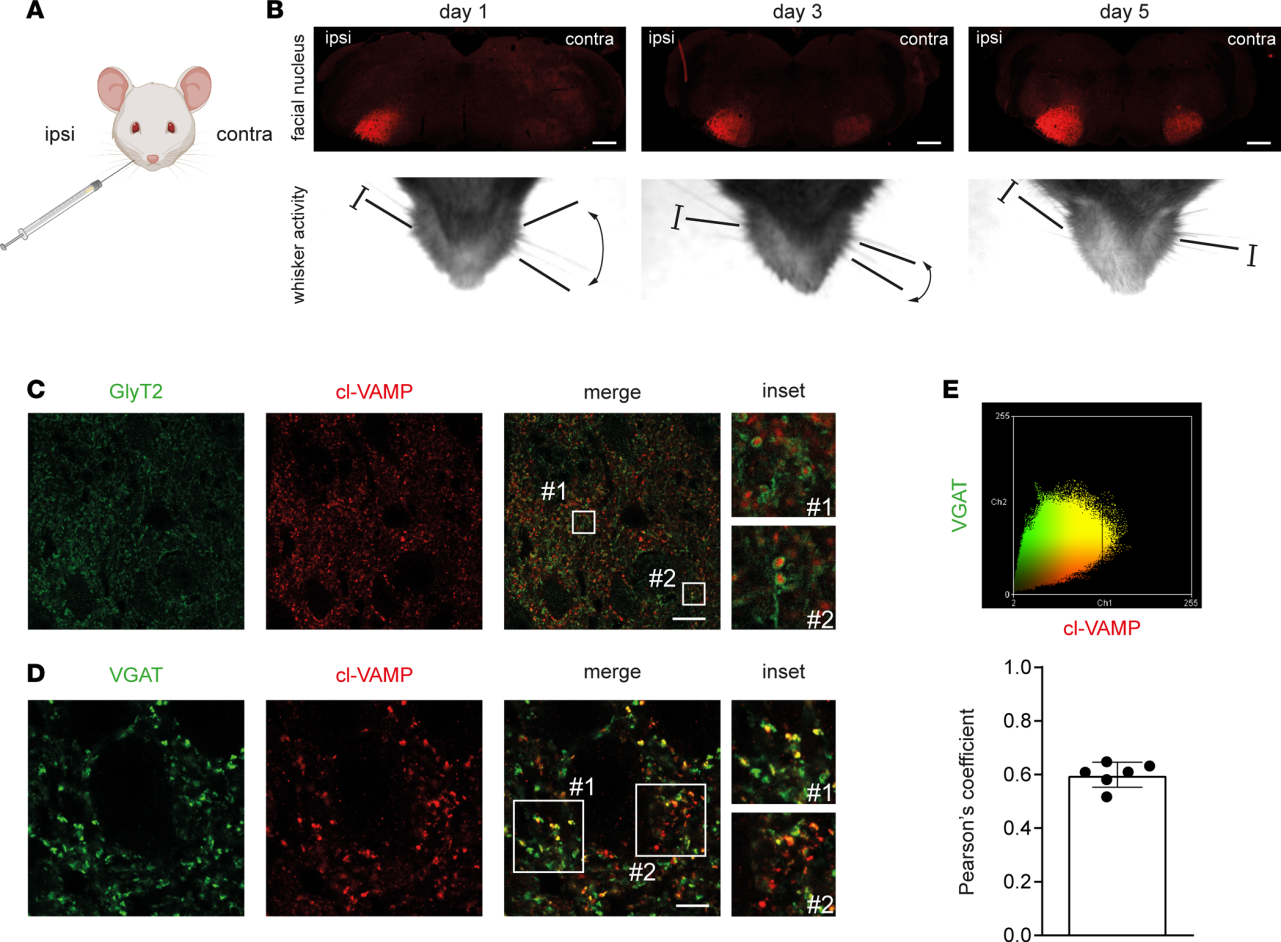
TeNT activity in the brainstem after injection in the WP is found at the level of inhibitory axon terminals. (**A**) Cartoon showing TeNT injection (1 ng/kg in a final volume of 1 μL) in the WP. (**B**) TeNT activity causes the appearance and progressive accumulation of cl-VAMP (red) in the facial nucleus (FN), which acts as a reporter to illuminate the brainstem areas reached by the toxin. As soon as 1 day after injection, the ipsilateral FN displays a strong signal of cl-VAMP (upper panels), which increases over time, though the mice still have flaccid paralysis (bottom panels). From day 3, a faint signal appears also in the contralateral side, when the noninjected WP starts to be spastic, and becomes clearly stained at day 5, when the spasticity of the noninjected WP is fully attained; scale bars, 500 μm. (**C**) The signal of cl-VAMP (red) is surrounded by the staining of GlyT2 (green), the plasma membrane transporter involved in the reuptake of glycine in the synaptic cleft, indicating that TeNT mainly acts within the presynaptic cytoplasm of inhibitory interneurons; scale bar, 25 μm. The insets show a 10× original magnification. (**D**) The signal of cl-VAMP (red) appears as puncta and colocalizes with the vesicular transporter of GABA and glycine (VGAT, green), indicating that TeNT activity occurs specifically at the level of synaptic vesicles within inhibitory axon terminals; scale bar, 10 μm. The insets show a 3× original magnification. (**E**) Pearson’s colocalization analysis between cl-VAMP (red) and VGAT (green) signals shown as a scatterplot (top panel) and as a histogram of the correlation coefficient (bottom panel). Black circles indicate the number of brainstem slices used for the analysis.

The above results account for the molecular origin of CT facial palsy. Yet, the cardinal and most dangerous symptom of CT is the spastic paralysis of the head and facial muscles, rapidly followed by dysfunction of swallowing, respiration, and heart function (17, 18, 21). Accordingly, the brainstem areas corresponding to these essential physiological functions, suspected to be affected by TeNT proteolysis, were studied by monitoring VAMP cleavage as a function of time after TeNT inoculation in the WP (Figure 4A). As soon as 1 day after injection, a strong signal of cl-VAMP appeared at the level of the ipsilateral FN containing the motor efferents of the whisking musculature (Figure 4B) (31, 32). Consistent with a presynaptic action within inhibitory interneurons, we found the staining of GlyT2, the presynaptic plasma membrane transporter of glycine, around the cl-VAMP signal (Figure 4C), which appeared as puncta colocalizing with the signal of an antibody specific for VGAT, the vesicular transporter of GABA and glycine (Figure 4D). This staining suggests that VAMP cleavage occurred within the presynaptic space of axon terminals of inhibitory interneurons, where VAMP is localized on synaptic vesicles. The colocalization between cl-VAMP and VGAT was extensive but not complete (Figure 4E), yet some cl-VAMP puncta were not associated with this marker of inhibitory interneurons. This finding indicates that TeNT could also enter in the presynaptic space of non-glycinergic and non-GABAergic neurons, whose origin and contribution to the development of tetanus spasticity remain to be established.

With time, the intensity and occupancy of the cl-VAMP signal in the FN progressively increased, and some staining started to be visible by day 3 also in the contralateral FN. Of note, such a faint signal (compared with ipsilateral FN) was sufficient to cause muscle spasticity in the contralateral (noninjected) WP and, similarly, at day 5, suggesting that TeNT-induced muscle spasticity is determined by a comparatively limited amount of VAMP cleaved. Accordingly, considering the strong cl-VAMP signal in the ipsilateral FN, we postulated that the effect of TeNT at the NMJ causing the nerve palsy is dominant on the central activity on inhibitory interneurons associated with muscle spasticity.

### TeNT central activity diffuses throughout brainstem nuclei, causing respiratory dysfunction before systemic spasticity.

**Figure 5:**
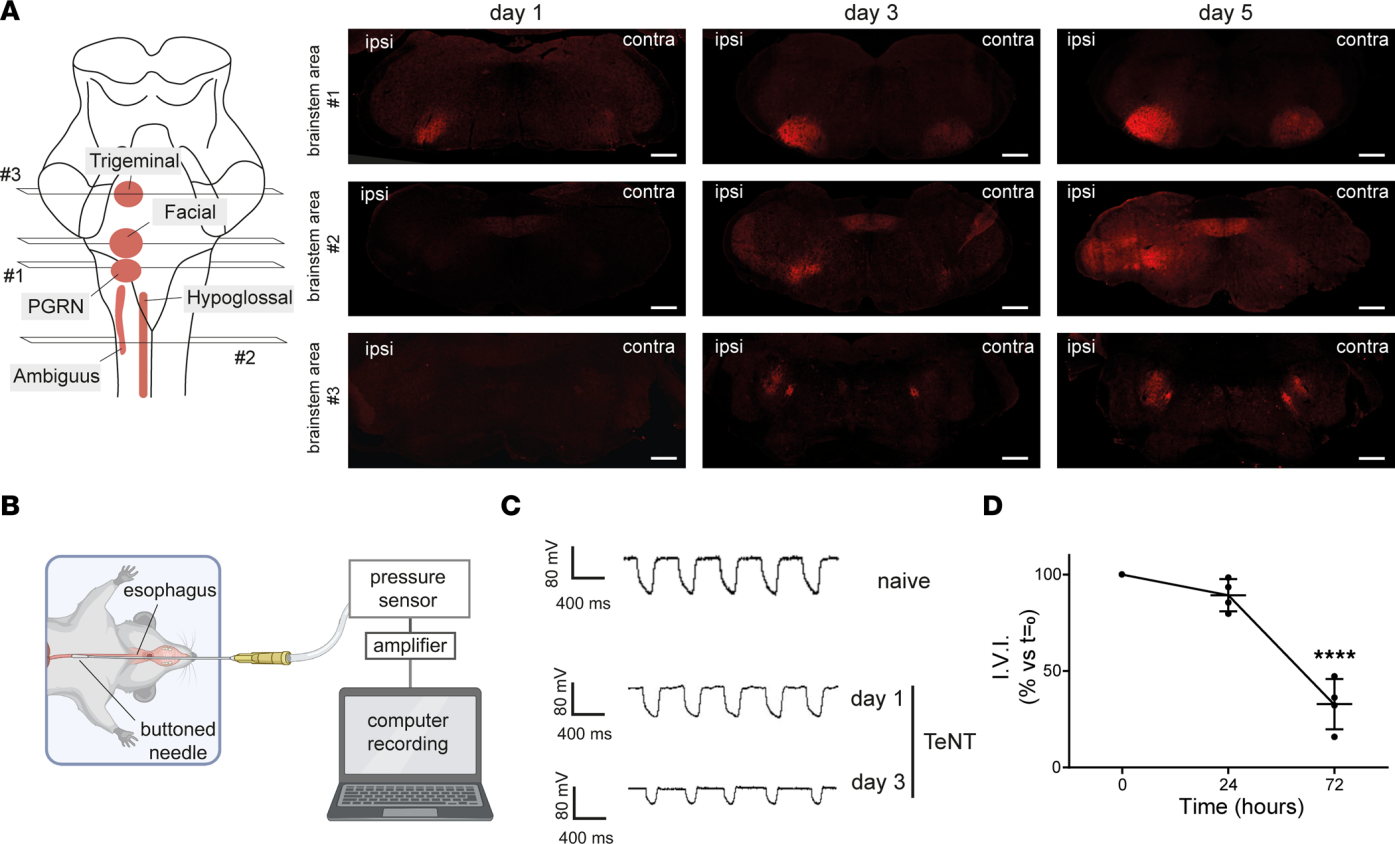
TeNT activity in the brainstem after injection in the WP rapidly spreads to nuclei controlling vital functions, including respiration. (**A**) TeNT was injected in the left WP (1 ng/kg in a final volume of 1 μL) that caused the appearance of cl-VAMP (red) at the level of different brainstem areas: by day 1 the paragigantocellular reticular nucleus (PGRN), involved in the regulation of respiratory and autonomic cardiovascular functions, and by day 3 trigeminal motor (TM), hypoglossal (HN), and ambiguus (NA) nuclei, controlling mastication, swallowing, and the upper airways (larynx and pharynx), respectively; scale bars, 500 μm. (**B**) Scheme illustrating the experimental setup used to measure the intraesophageal pressure in living mice, which provides an accurate air volume exchanged by the animal during the respiratory cycle: a buttoned needle connected to a pressure sensor is inserted in the mouse esophagus to measure the pressure; the signal is amplified and digitalized by computer. (**C**) Respirograms from naive (top trace) and TeNT-treated mice 1 day (central trace) and 3 days (bottom trace) after WP injection. Each trace deflection reports the pressure variations occurring during a single respiratory act, which highlight the progressive reduction in the air volume exchanged during CT; 1 day after TeNT, when VAMP cleavage is confined in the FN, few, if any, changes are present compared to naive respiration; at day 3 deflections at each respiratory act appeared markedly reduced, suggesting a deterioration in the ability of the mouse to breathe. (**D**) Quantification of the respiratory ability reported as “inferred ventilation index” (IVI), calculated as the overall volume of air exchanged by the animal over 20 seconds (see Methods); means ± SD; ****P < 0.0001 assessed by 1-way ANOVA with multiple comparisons and Bonferroni’s test. The analysis was done with 4 animals per time point.

On day 1, VAMP cleavage was mainly confined in the ipsilateral FN, but Figure 5A shows a weak staining also in the paragigantocellular reticular nucleus (PGRN), a brainstem area located caudally just behind the FN containing neuronal nuclei involved in the control of respiration and autonomic cardiovascular functions (Figure 5A) (33, 34). Moreover, by day 3 VAMP cleavage was detected also in the trigeminal motor (TM), hypoglossal (HN), and ambiguus (NA) nuclei, i.e., brainstem areas controlling mastication, swallowing, and more broadly the activity of the upper respiratory tract (larynx and pharynx). Of note, cl-VAMP staining markedly increased at day 5 in all these nuclei, but not elsewhere, suggesting that TeNT diffusion within the brainstem remained localized and specific.

Given that the PGRN, the HN, and the NA are involved in the control of the upper airways’ function and of respiration, we wondered whether TeNT action in these nuclei could cause any change in breathing. To answer this question, we took advantage of an electrophysiological assay that allows one to measure the intraesophageal pressure in living mice (Figure 5, B and C); this provides an accurate estimation of the intrapleural pressure, and thus, indirectly, of the air volume exchanged by the animal during the respiratory cycle. Of note, this technique is minimally invasive, allowing repeated measurements in the same animal, before and after toxin injection (35).

Figure 5C shows the normal respirogram of a mouse before toxin treatment. One day after TeNT injection in the WP, when the cleavage of VAMP is confined in the FN, we detected little, if any, change in the mouse respirogram. Conversely, when TeNT activity spread to PGRN, HA, and NA at day 3, the variations of intraesophageal pressure at each ventilation act were markedly reduced, consistent with a defect in the mouse’s ability to breathe. To provide a quantitative estimation, we calculated an “inferred ventilation index” (IVI), i.e., a parameter indicative of the overall volume of air exchanged by the animals over 20 seconds. As shown in Figure 5D, at day 1 IVI was comparable to that of naive mice, while it decayed to about 40% at day 3, indicating a pronounced reduction in ventilation, though the animal had not yet developed evident symptoms of tetanus.

Together, these data suggest that when TeNT reaches the central nervous system, it first affects inhibitory interneurons impinging on the motor efferents responsible for its retroaxonal transport, but then it traffics trans-neuronally to adjacent areas involved in the control of mastication, swallowing, and respiration, causing a respiratory deficit without systemic spasticity, as occurs in human CT (17, 18, 20, 22).

### TeNT spreading in the brainstem depends on both peripheral and brainstem intraparenchymal diffusion.

Intrigued by the rapid spreading of the cl-VAMP signal in the brainstem, we wondered how TeNT can diffuse to several groups of neurons after having been taken up by neuronal efferents innervating head muscles.

**Figure 6:**
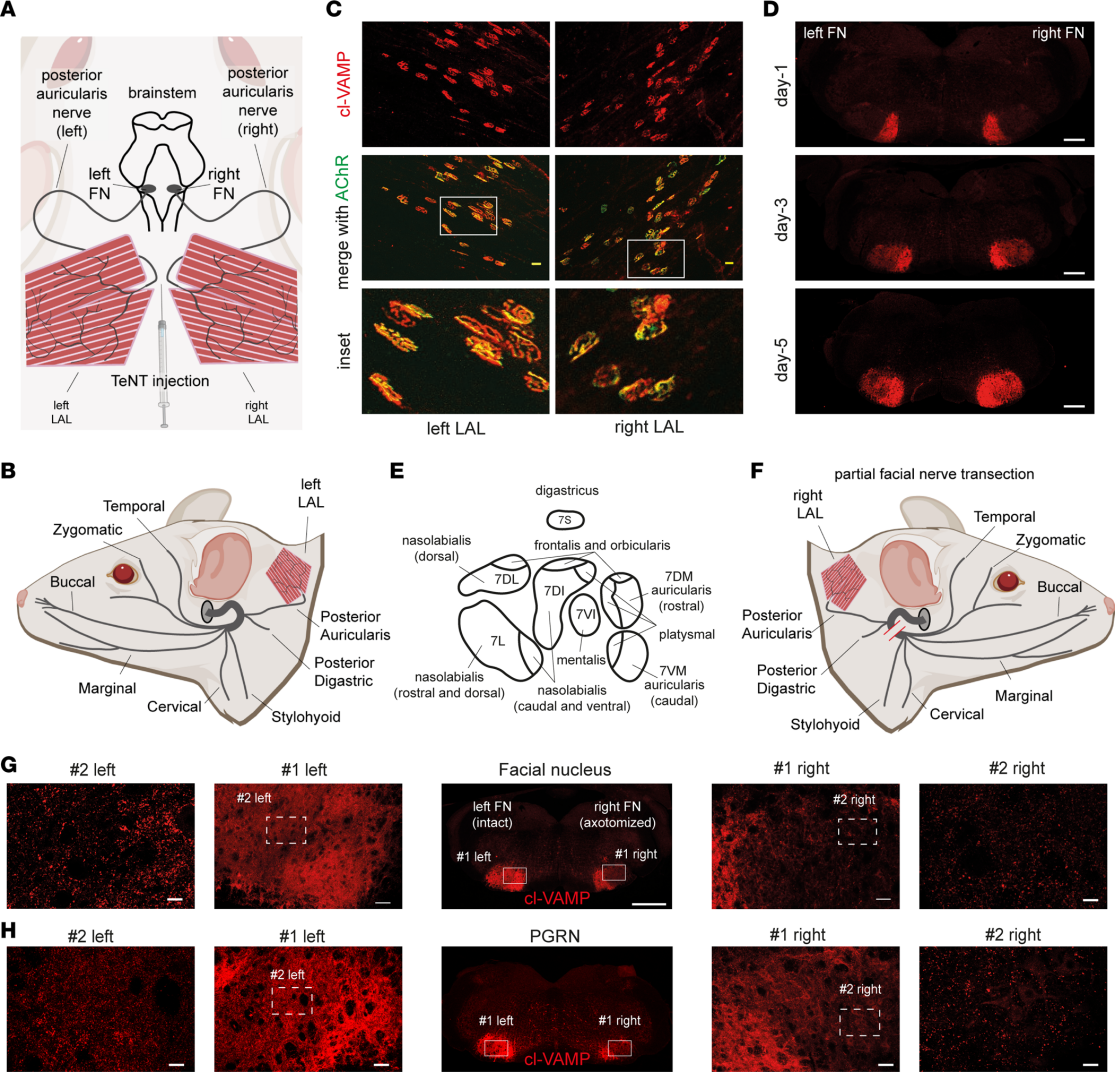
A combination of peripheral diffusion and intraparenchymal dissemination causes the rapid spreading of TeNT activity among brainstem neurons. (**A**) Scheme showing the bilateral innervation of LAL muscles by the posterior auricularis branch of the facial nerve connecting LAL NMJ motoneuron somas residing in the FN. (**B**) Scheme of facial nerve innervation of the dermomuscular system of the mouse head; each facial nerve exits at the level of the stylomastoid foramen (gray circle) and splits into distinct branches. (**C**) TeNT injection (1 ng/kg in 5 μL) between the 2 LAL muscles causes the appearance of cl-VAMP (red) in both left and right LALs; AChR labeling (green) with fluorescent α-bungarotoxin shows the NMJs. (**D**) TeNT injection between the 2 LAL muscles causes the bilateral appearance of cl-VAMP (red) in both FN; at day 1, the signal is restricted to the medial portions and then spreads distally, affecting the entire FN at day 5. (**E**) Scheme of facial motor subnuclei with efferent to specific muscles of the head. (**F**) Scheme of right facial nerve transection (red bars) showing the disconnection of all facial nerve branches except posterior auricular and digastric. (**G** and **H**) TeNT injection between the 2 LAL muscles with partial transection of the right facial nerve causes at day 5 a different distribution of cl-VAMP (red) in the FN (**G**) and PGRN (**H**) (central panels, scale bar: 500 μm): the nonaxotomized FN and PGRN display a signal diffused throughout the entire nucleus; cl-VAMP in the axotomized FN mainly affects the auricular and digastric subnuclei. Cl-VAMP also appears in distal FN subnuclei and PGRN like puncta around neuron somas, as shown via the progressive magnifications (scale bars: #1 = 50 μm; #2 = 20 μm). Images are representative of 1 of 3 animals.

The observation that TeNT injection in one WP causes VAMP cleavage in both ipsi- and contralateral brainstem areas indicates that the toxin partly diffuses at the level of peripheral tissues. On the other hand, the detection of VAMP cleavage within nuclei like the PGRN, which have no sensorimotor efferents projecting to peripheral tissues, suggests that TeNT could undergo intraparenchymal dissemination after it arrives in the brainstem. To test this possibility, we exploited the particular anatomy of the 2 facial nuclei and set up an experiment on the levator auris longus (LAL) muscles, 2 muscles of the mouse pinna used to move the ears. As shown in Figure 6A, each of the 2 LALs is innervated by the posterior auricularis nerve, i.e., 1 out of the several branches of the FN (Figure 6B). Upon TeNT injection between the 2 LALs, we induced a bilateral intoxication of both muscles that was accompanied by a clear signal of cl-VAMP in their NMJs (Figure 6C). In addition to showing that TeNT peripheral effect was not limited to the WP, this procedure allowed TeNT retroaxonal transport to the brainstem via the 2 facial nerves and, as a result, elicited a simultaneous bilateral cleavage of VAMP in the 2 facial nuclei (Figure 6D). Also in this case, cl-VAMP initially (day 1) appeared in subnuclei of the FN populated by motoneuron efferents of the auricularis nerve (32) but then progressively spread and reached all the areas populated by motoneuron efferents of the entire mouse snout (Figure 6E) (32). Accordingly, we performed a partial transection to disconnect all FN efferents except those of the posterior auricularis (and digastric) subnuclei (Figure 6F), and then TeNT was injected between the LALs; its activity in the FN was monitored at day 5 via VAMP cleavage. As shown in Figure 6G, the axotomized FN displayed strong staining of cl-VAMP only in posterior auricularis and digastric subnuclei, while the FN with the intact nerve showed cl-VAMP appearance in the whole FN. However, a more careful inspection revealed cl-VAMP staining within axotomized subnuclei, though less intense compared with the contralateral nonaxotomized FN. In particular, the signal appeared more intense in proximal areas, especially at the level of platysmal subnuclei, and still detectable also in more distant subnuclei, where it appeared as discrete puncta around motoneuron soma. Intriguingly, we found a similar scenario also at the level of the PGRN, which showed a clear staining for cl-VAMP notwithstanding the partial axotomy of the facial nerve (Figure 6H). Considering that the PGRN does not have efferents reaching peripheral tissues but has internal connections with the FN (36), this result strongly suggests that the spread of TeNT activity into the brainstem, in addition to peripheral diffusion, also derives from intraparenchymal diffusion of the toxin.

## Discussion

The present study unravels the pathogenesis and contrasting symptoms of CT and discloses potentially novel activities of TeNT within the central and peripheral nervous systems. These findings were made possible by developing a model of CT based on the local injection of TeNT in the mouse head muscles and by using an antibody that recognizes only VAMP cleaved by tetanus neurotoxin (23).

The first major finding is the unexpected activity of TeNT at the NMJ of facial muscles. Together with the electrophysiological analyses showing impaired NMJ neurotransmission, which extend previous electromyographical findings in patients (37, 38), the demonstration of TeNT cleavage of VAMP within facial NMJs, obtained here for the first time to our knowledge, discloses the molecular lesion at the basis of CT facial palsy. This symptom is a main confounding factor for CT diagnosis and is hardly associable with tetanus since TeNT toxic activity is traditionally considered to affect exclusively neurons of the spinal cord that lead to muscle contractures and spasms. Of note, TeNT local activity at the NMJ appears to be reversible. Although we did not investigate the molecular mechanism responsible for the functional recovery, it is likely that a major determinant of the persistence of TeNT paralytic action at the NMJ is the lifetime of its catalytic domain within the motor axon terminals, as is the case for botulinum neurotoxins (26, 27).

A second major finding is the rapidity of TeNT spreading within the brainstem as a result of both peripheral uptake and intraparenchymal dissemination. Notably, the combination of these 2 processes causes a broad and efficient intoxication of key neurons that control essential physiological functions, including breathing. This explains why a) TeNT displays its maximal toxicity in the brainstem (39), b) CT is a highly dangerous form of tetanus, and c) patients with CT suddenly and rapidly worsen after the onset of head muscle spasticity. In addition, these results clarify why CT can be very severe even without evolving into generalized tetanus (2, 40).

Whether the tropism for the brainstem derives from a particular affinity of TeNT for cranial nerve terminals remains to be established. Similarly, how TeNT intraparenchymal dissemination occurs, either via simple diffusion or via interneuronal consecutive cycles of retrograde transports as found for BoNT/A (41, 42), or their combination, remains unclear. Yet, connectome data show that the FN has inputs from the ipsilateral HN, input and output from the NA, and projections to PGRN and TM nucleus (36, 43). It is tempting to speculate that TeNT transneuronal trafficking privileges retroaxonal transport over the cytosolic entry also at central nerve terminals, thus supporting transnuclear spreading. Future investigations are necessary to reveal whether this mechanism contributes to the transition from local to generalized tetanus and to trismus being the initial symptom of tetanus (1–3, 15).

Another key observation of the present study is that the peripheral action of TeNT at the NMJ is dominant with respect to the activity of the toxin on inhibitory interneurons in the brainstem. This explains why nerve palsy in human CT can persist as a unique symptom for several days before head muscle spasticity, which then manifests suddenly and progresses to life-threatening symptoms in a short time (17, 20–22). Indeed, the peripheral effect first affects the muscles around the TeNT release site overshadowing the onset of spasticity; meanwhile, the toxin has the time to spread and intoxicate large portions of the brainstem. Arguably, this is the culprit factor responsible for the delay in CT diagnosis and the ensuing fast deterioration of patients’ conditions requiring intensive care (20–22).

In conclusion, the findings of the present paper suggest that patients presenting with an idiopathic facial nerve palsy should be immediately considered for a diagnosis of CT and accordingly treated with anti-TeNT immunoglobulin, when skin, gingival, or inner ear lesions are present. This procedure is well established, innocuous, and inexpensive, yet it is capable of preventing the nefarious consequences of tetanus. In light of this, purified monoclonal antibodies with high neutralization activity injected intrathecally in the cerebrospinal fluid in the brainstem could represent a strategy with even better therapeutic outcomes than the intramuscular one (8, 44).

## Methods

### Antibodies, reagents, and toxins.

TeNT was purified from *C*. *tetani* Harvard strain cultures and was kept at –80°C (45). When injected in vivo, the toxin was dissolved in physiological solution plus 0.2% gelatin (G2500, MilliporeSigma). An affinity-purified antiserum specific for TeNT-cleaved VAMP was obtained as recently described (23); anti-VAChT (1:500, 139 105), anti-intact VAMP-2 (1:500, 104 211), and anti-VGAT (1:500, 131 308) antibodies were purchased from Synaptic System; anti-GlyT2 (1:500, AB1773) was purchased from Chemicon; and α-bungarotoxin Alexa Fluor 488 conjugated (1:200, B13422) and anti–guinea pig Alexa Fluor 488 conjugated (1:200, A11073) were purchased from Thermo Fisher Scientific. Anti-rabbit Alexa Fluor 555 conjugated (1:200, A21428) was purchased from Life Technologies.

### Ventilation recordings.

Recordings were performed before and 24 and 72 hours after intoxication with 1 ng/kg of TeNT (diluted in 3 μL physiological solution containing 0.2% gelatin). Animals were anesthetized (xylazine/zoletil 48/16 mg/kg). A bottomed plastic feeding tube (20 ga × 38 mm, Instech Laboratories) was carefully introduced into the oral cavity and placed in the esophagus at the level of the mediastinum. Mice were laid on the left side on a prewarmed heat pad. Pressure variations were recorded via a pressure sensor (Honeywell, 142PC01D) connected to an amplifier. Traces were digitized with WinEDR V3.4.6 software (Strathclyde University) and analyzed with Clampfit (Axon). We inferred the volume of exchanged air by measuring esophageal pressure variations, which reflect intrapleural pressure variations (46). At least 120 epochs were recorded, and at least 20 epochs were used for the analysis at each time point. The IVI parameter was calculated for each animal as the product of the mean area of the peaks multiplied by the number of peaks within 20 seconds. Data represent the percentage of *t*_0_ taken in the same animal.

### Whisking behavior.

Whisking behavior in mice was recorded in awake individuals head-fixed with a custom-made head plate implanted onto the skull. Briefly, animals were anesthetized (isoflurane, Abbott Laboratories), then laid on a heating pad, and eye drying was avoided with an ophthalmic solution. The scalp was shaved, locally anesthetized with 2.5% lidocaine, and disinfected with betadine solution. An incision was made to expose the skull, and the head plate was fixed with dental cement. Baytril was administered to prevent infection, and the animal was allowed to recover in a warmed clean cage under monitoring to exclude signs of pain or distress. After animals recovered from the surgery (2–3 days), they were habituated to head restraint for 1 week by time-increased sessions each day (47) in the setup consisting of a high-speed camera (acA800-510um, Basler) and custom-made infrared illumination. Videos were recorded at 300 Hz for 2 minutes, taken before and at indicated times after TeNT injection.

For rat experiments, TeNT injections (50 pg in 0.9% NaCl 0.2% gelatin) in the WP were done under anesthesia with isoflurane. Whisking behavior was recorded with a GoPro 10 camera at 240 fps frame rate and 1,920/s shutter speed and evaluated by monitoring off-line the videos frame by frame to spot the points of maximum extension and retraction of vibrissae.

### CMAP electromyography.

Animals were injected in the WP with the indicated amount of TeNT (diluted in 0.9% NaCl, 0.2% gelatin, 1 μL of volume) or vehicle only. At indicated times, the animals were anesthetized (xylazine/zoletil 48/16 mg/kg), and CMAP was evoked by supramaximal stimulation with an S88 stimulator connected to needle electrodes (Grass) placed near the nerve. Recording and reference electrodes (Grass) were inserted into the WP and under the skin at the nose tip, respectively. The ground electrode was placed subcutaneously in the back lumbar area. Signals were digitized with an A/C interface (National Instruments) and then fed to a PC for online visualization (WinEDR) and software analysis (pClamp). CMAPs were determined as average peak-to-peak intensity (in mV) from 5 supramaximal rectangular stimulation pulses (200 μs) delivered from the isolated stimulator via a 2-channel amplifier (Npi Electronic). Stimulation and recording were controlled by a PC with Spike 2 software and a Micro1401-4 control panel (CED).

### Immunofluorescence.

WP and LAL muscles from CD1 mice or WPs from rats were dissected at indicated time points and fixed (4% paraformaldehyde, 30 minutes, room temperature [RT]). Brainstems were fixed by intracardial perfusion, collected, postfixed overnight (4% paraformaldehyde, 15% sucrose), and then left for at least 2 days in PBS 30% sucrose. Brainstem and WP slices of 30 μm of thickness were cut with a cryostat (Leica), while LALs were used for whole-mount staining. Tissues were quenched in PBS 0.25% NH_4_Cl for 20 minutes, permeabilized, and saturated for 2 hours in blocking solution (15% goat serum, 2% BSA, 0.25% gelatin, 0.20% glycine, 0.5% Triton X-100 in PBS), then incubated with primary antibodies for 24 hours (slices) or 72 hours (LAL) in blocking solution at 4°C. Muscles were then washed 3 times in PBS and incubated with secondary antibodies for 2 hours at RT. Images were collected with a confocal microscope (Zeiss LSM900 Airyscan2) equipped with N-Achroplan (5×/0.15 Ph1 air), EC Plan-Neofluar (20×/0.5 air or 40×/0.45 oil) or Plan-Apochromat (100×/1.4 oil) objective. Laser excitation, power intensity, and emission range were kept constant and set to minimize bleed through.

The colocalization analysis was performed with ImageJ (plug-in colocalization analysis; NIH) on maximal projections of confocal images from at least 3 randomly chosen areas in at least 3 brainstem slices of the FN.

### Statistics.

Sample sizes were determined by analysis based on data collected by our laboratory in published studies. We used at least *n* = 4 mice/group for all experiments. We ensured the blinded conduct of experiments. Data were displayed as means ± SD, calculated with GraphPad Prism. Statistical significance was evaluated using unpaired 2-tailed Student’s *t* test or by 1-way ANOVA. *P* < 0.05 was considered statistically significant.

### Study approval.

Our studies were carried out in accordance with the European Community Council Directive number 2010/63/UE and with national laws and policies after approval by the local authority veterinary services of the University of Padua and the University of Zagreb.

Mice were purchased from Charles River Laboratories Italia and maintained under 12-hour light/12-hour dark cycle in a controlled environment with water and food ad libitum. Rats (350–400 g) were purchased from Inotiv and kept 2 to 3 per cage in a controlled environment with a 12-hour light/12-hour dark cycle at 21°C to 23°C and 40% to 70% humidity. Food pellets and water were available ad libitum.

### Data availability.

See Supporting Data Values.

## Author contributions

CM, OR, and MP conceived the study; FF, SV, MT, PŠ, PM, and IM investigated; FF, SV, MT, PŠ, PM, IM, and AM curated data; CM, MP, AM, MC, and OR supervised; CM and MP wrote the original draft; and all authors reviewed and edited the manuscript.

## Supplementary Material

Supplemental data

Supplemental video 1

Supplemental video 2

Supplemental video 3

Supplemental video 4

Supplemental video 5

Supplemental video 6

Supplemental video 7

Supplemental video 8

Supplemental video 9

Supporting data values

## Version 1 05/09/2023

In-Press Preview

## Version 2 06/08/2023

Electronic publication
