# Supplementary material for: Facial neuromuscular junctions and brainstem nuclei are the target of tetanus neurotoxin in cephalic tetanus
Source: JCI Insight. 2023 Jun 8;8(11):e166978. doi: 10.1172/jci.insight.166978 (PMC10393225; doi:10.1172/jci.insight.166978)
Supplement: Supplemental data [file jciinsight-8-166978-s021.pdf]

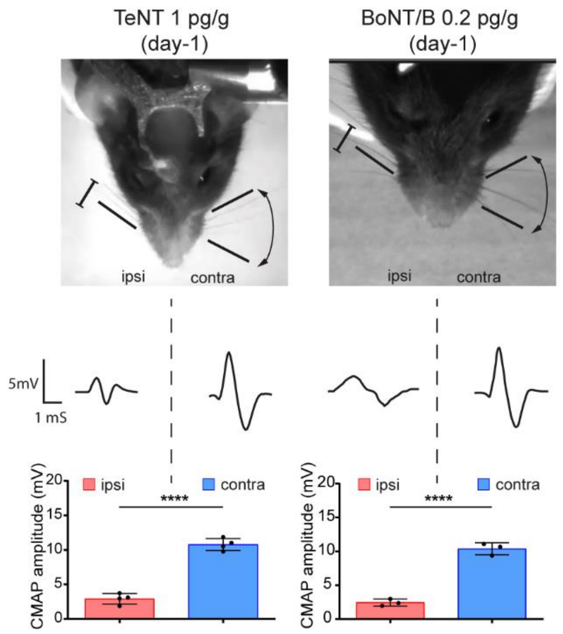

Supplementary Figure 1. TeNT causes a flaccid paralysis similar to BoNT/B upon local injection in the WP. Representative video frames showing the whisking ability in mice one day after TeNT (left) or BoNT/B (right) inoculation in injected (ipsi) and non-injected (contra) WPs; black arrows and bars indicate the movement ability of the vibrissae as deduced from recorded videos; segments with blunt ends indicate full paralysis. The traces indicated the CMAP recordings in ipsi and contra WPs one day after the injection of the toxins and their quantifications are shown in bottom graphs. Data are expressed as means ± s.d.; P values (****<0.0001) assessed by t student test. Black circles indicate the number of animals used in the experiment.

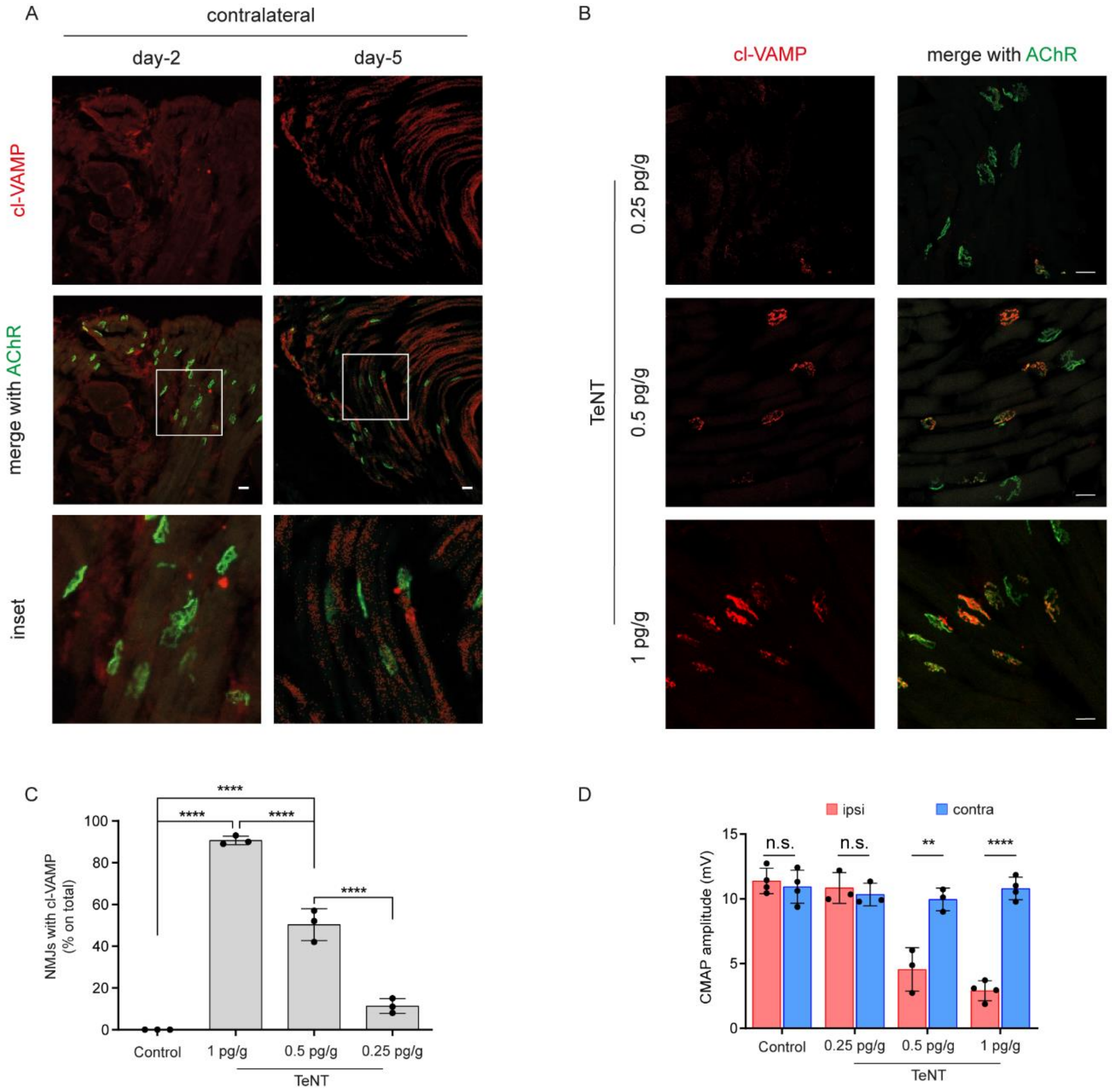

Supplementary Figure 2. TeNT causes a flaccid paralysis similar to BoNT/B upon local injection in the WP. A) Confocal images of the contralateral WP musculature from mice treated with 1 pg/g TeNT at indicated times after injection; the red signal indicates the cleavage of VAMP at the NMJ identified through the labeling of nicotinic acetylcholine receptors (AChR, green) with fluorescent α-bungarotoxin; insets show a magnification. Images are representative of one from several independent experiments; scale bars, 50 µm. **B)** Confocal images showing the signal of cl-VAMP (red) one day after the injection of the indicated doses of TeNT in the WP. Nicotinic acetylcholine receptors (AChR, green) are labeled with fluorescent α-bungarotoxin. Scale bar, 50 µm. **C)** Quantification of the number of NMJs displaying the cl-VAMP signal one day after the injection of the indicated doses of TeNT in the WP. Data are expressed as means ± s.d.; P values (****<0.0001) assessed by one way ANOVA with Bonferroni test. Black circles indicate the number of animals used in the experiment. **D**) Quantification of the CMAP amplitude in the ipsilateral (red) and contralateral (blue) WPs one day after the injection of the indicated doses of TeNT in one WP. Data are expressed as means ± s.d.; P values (****<0.0001) assessed by t student test. Black circles indicate the number of animals used in the experiment.
